# Identifying Non-Math Students from Brain MRIs with an Ensemble Classifier Based on Subspace-Enhanced Contrastive Learning

**DOI:** 10.3390/brainsci12070908

**Published:** 2022-07-12

**Authors:** Shuhui Liu, Yupei Zhang, Jiajie Peng, Tao Wang, Xuequn Shang

**Affiliations:** 1 School of Computer Science, Northwestern Polytechnical University, Xi’an 710072, China; lsh@mail.nwpu.edu.cn (S.L.); jiajiepeng@nwpu.edu.cn (J.P.); twang@nwpu.edu.cn (T.W.); shang@nwpu.edu.cn (X.S.); 2 Key Laboratory of Big Data Storage and Management, MIIT, Xi’an 710129, China

**Keywords:** brain science, neuroscience, contrastive learning, MRIs identification

## Abstract

In current research processes, mathematical learning has significantly impacted the brain’s plasticity and cognitive functions. While biochemical changes in brain have been investigated by magnetic resonance spectroscopy, our study attempts to identify non-math students by using magnetic resonance imaging scans (MRIs). The proposed method crops the left middle front gyrus (MFG) region from the MRI, resulting in a multi-instance classification problem. Then, subspace enhanced contrastive learning is employed on all instances to learn robust deep features, followed by an ensemble classifier based on multiple-layer-perceptron models for student identification. The experiments were conducted on 123 MRIs taken from 72 math students and 51 non-math students. The proposed method arrived at an accuracy of 73.7% for image classification and 91.8% for student classification. Results show the proposed workflow successfully identifies the students who lack mathematical education by using MRI data. This study provides insights into the impact of mathematical education on brain development from structural imaging.

## 1. Introduction

Education, always a significant activity in human development, has a long-term impact on an individual’s career and life [1]. As one of the most concerning items, mathematical education has been associated with many quality-of-life and development indices, including financial stability, mental, and fertility [2]. Therefore, there has been significant interest in the research on mathematical education and yielded a wide range of education discoveries and educational tools from biological function to artificial intelligence (AI) [3,4,5]. As described in [6], translational medicine (TM) is not only the application of research discoveries into clinical practice but also the transformation of the entire medical model, which ultimately improves the overall medical level and helps patients solve health problems. In the research of mathematics education, a major objective is not simply to understand and recognize the brain structure pattern or biomolecular process but to help people to have a clearer understanding of themselves, find the field of interest, improve and enhance their quality of life. However, the basic research on mathematics education is still in the exploratory stage. And it still needs to use advanced data mining technology to mine knowledge from medical images and biological molecular data to reveal the related mechanism of phenotype.

This paper summarized these related studies into biological analysis, psychological measurement, and data information. The biological research aims to understand the biochemical association between biology and education, e.g., the impact of education on the brain, by using statistical analysis tools [3,7,8]. Brain et al., reviewed the studies of specific learning disabilities to understand the complex etiology, co-occurrences. Accordingly, they underpinned the training of teachers, school psychologists, and clinicians on the optimization of learning contexts for individual learners [9]. By investigating 27 studies on numerical cognition in the living brain, Korbinian et al., arrived that numerical cognition is subserved by a frontoparietal network that connects the cortex, basal ganglia, and thalamus [10]. Annie et al., explored the association between neural changes occurring during adolescence and cognitive functions and behaviors, suggesting teachers could help students remedy students’ misconceptions in science and mathematics [11]. While biological analysis focuses on brain development, psychological measurement is to understand the education process from sociality and mentality by seeking parameters for cognitive models, e.g., item response theory [4,12]. Robert et al., explored the nature of the relations among prior achievement, self-efficacy, outcome expectations, and interests in students’ choice and performance for mathematics-related college courses, showing the potential effectiveness of the social cognitive theory [13]. Leslie reviewed the studies from 1901 to the present and augmented that the mathematics curricula should be constructed following children’s psychology [14]. Yupei et al., developed the model of item response theory to predict student responses to the following questions by training the latent factor model on response records [4,12]. Under popular data science and technology, [14,15], AI-aided education tools and education discovery are becoming hot study fields, e.g., educational data mining (EDM) and learning analytics (LA) [4,5,12,16]. Natalia et al., presents a study of cognitive test anxiety and self-perception through questionnaires from over 2000 primary school students and 200 teachers, showing girls are more likely to experiment with a negative attitude toward mathematics than boys in Spain [17]. Yue et al., proposed a self-placed graph memory network to predict the student’s Grade Point Average (GPA) while finding the abnormal student [16]. By investigating students’ knowledge state, Yupei et al., proposed a meta-knowledge learning model that aims to learn the latent meta knowledge instead of the manual Q-matrix [4]. Among these research works, the investigation of the neural substrates of mathematical cognition and education provides the biological perspective to the impact of mathematical education [3].

In recent years, many works studied the impact of mathematical education on brain regions via the technique of neuroimage [3,18]. Marie et al., employed quantitative meta-analyses of fMRI studies to identify brain regions concordant among studies on number and calculation, resulting in a topographical brain atlas of arithmetic [18]. Mariano et al., presented four specific cases in which neuroscience synergized with other disciplines to serve the education and argued that the neuroscience method could broaden our understanding of education [19]. Marie et al., showed brain activity in parietal and frontal cortices, core areas related to mental-arithmetic, as well as brain regions, served for mathematical-related problem-solving, leading to a topographical atlas of mathematical processes in children [20]. To investigate the impact of a lack of mathematical education on adolescent brain development and future attainment, George et al., acquired fMRIs from more than 120 individuals composed of math or non-math students [3]. They found the γ-aminobutyric acid (GABA) concentration within the middle frontal gyrus (MFG) successfully classified math or non-math students and the adverse effects on brain plasticity and cognitive functions due to a lack of mathematical education. However, few studies investigated the impact of education from structural images. Medical images could present the brain structure, which is often used for disease diagnosis and therapy [21,22]. In addition, the quality of data labels is essential for supervised models. Acquiring high-quality data labels requires experienced experts to annotate the data in biomedical imaging. However, the quality of the data labels usually needs further testing. Contrast learning is self-supervised learning, which learns knowledge from unlabeled images and does not rely on labeled data.

In this paper, we made this attempt to identify non-math students from MRIs by using the popular deep feature learning technique [5,22]. Since the region of MFG has been associated with mathematic learning [3], we first cropped MFGs from MRIs to feed our deep contrastive model that is to implement robust feature learning [23]. After the feature learning, we built an ensemble classifier that is based on the multiple layer perception (MLP) to identify math or non-math students due to its multi-instance setting [24]. On 123 MRIs that were acquired from adolescents in the United Kingdom [3], composed of 72 math students and 51 non-math students, our method achieved an accuracy of 73.7% for image classification and 91.8% for student classification. On the image-level analyses of classification results, MRI slices have various difficulties, showing different impacts of math education in the MFG. Our study proves a path to understanding education by using brain images.

## 2. Materials and Methods

This study aims to identify math or non-math adolescent students by using MRI data to understand the impact of mathematical education on brain structure in the MFG region. To this end, we have the following study workflow: (1) acquiring MRIs from adolescent students that includes math students and non-math students and cropping all images into the MFG region [3]; (2) designing a classification tool by contrastive learning and ensemble learning [23,24], (3) evaluating the classification performance followed by several experiment analyses.

### 2.1. MRI Data and Preprocessing

**Figure 1:**
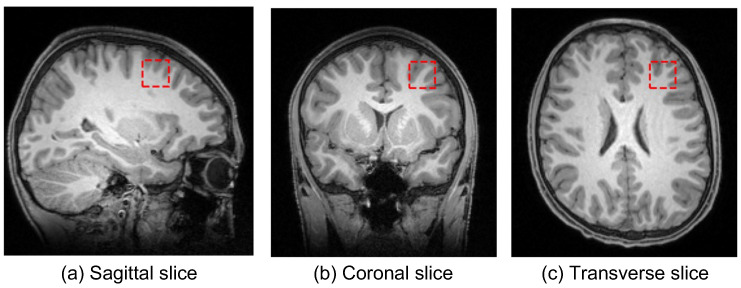
The T1-weighted MRI and the left MFG region. Three subplots are (**a**) a sagittal slice from left to right, (**b**) a coronal slice from top to bottom, and (**c**) a transverse slice from back to front, respectively.

In the United Kingdom, 16-y-old adolescents can choose to stop studying math as part of their advanced, i.e., A-level, subjects. Towards a single dependent variable in the study, a math group consisted of 72 students who engaged in A-level maths. A non-math group consisted of 51 students who were not engaged in A-level maths. The used 123 MRI data were acquired at the Oxford Centre for Function MRI of the Brain (FMRIB) on 3T Siemens MAGNETOM Prisma MRI System equipped with a 32-channel receive-only head coil. And anatomical high-resolution T1-weighted scans were acquired using an MPRAGE sequence consisting of 192 slices, where repetition time TR=1900 ms, echo time TE=3.97 ms, and voxel size=1×1×1 mm. The voxels of interest (VOI) of size 20×20×20 mm were manually centered in the MFG based on the individual’s T1-weighted images while the participant was lying down in the MR scanner [3]. Slices of the T1-weighted MRI and the left MFG region in three different directions were shown in Figure 1.

### 2.2. The Proposed Method

**Figure 2:**
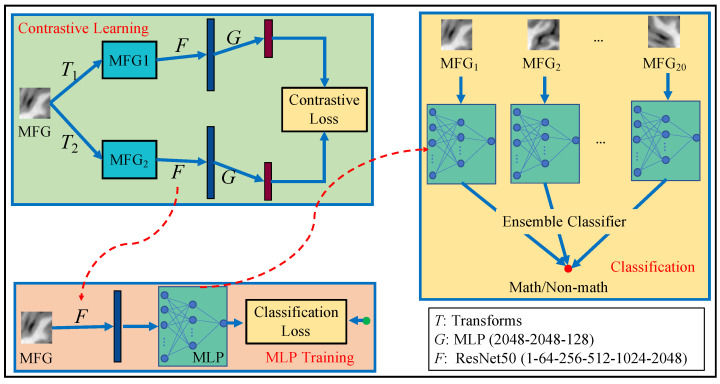
Our workflow. There are three steps, i.e., contrastive learning for deep features, MLP training for base classifiers, and Classification for combining multi-instance predictions.

The proposed method includes a feature learning stage and a classifier learning stage. Feature learning is to capture the intrinsic image representation by using the popular framework of contrastive learning, which is composed of ResNets and MLP [23]. The classifier learning stage trains an MLP for a multi-instance classification task and then ensemble all MLP results by simply voting. The main workflow of this study is shown in Figure 2. To make a clear statement, we here define the three tasks in our proposed framework.

**Definition** **1.** *Feature learning aims to transfer an MRI slice into a representation vector by employing the popular framework of contrastive learning [23], shown in Figure 2. The contrastive learning model gives rise to the mapping F for feature learning on MRI slices.*

**Definition** **2.** *Image classification aims to identify an MRI slice into the math class or the non-math class, implemented by training an MLP model shown in Figure 2. More specifically, the proposed method trains 20 MLPs, where $MLP_{i}$ is for these MRI slices with No. i*(i∈{1,2,⋯,20}).

**Definition** **3.** *Student classification aims to identify a student into the math class or the non-math class implemented by considering all 20 results of MRI image classification shown in Figure 2. More specifically, the 20 MLPs identify the 20 MRI slices and then vote for the student label.*

#### 2.2.1. Subspace-Enhanced Contrastive Learning

Contrastive learning (CL) is a recently proposed scheme for robust feature learning and has been already used in many studies, e.g., image classification [23], text classification [25], and medical image segmentation [26]. CL learns the intrinsic data representation by training a representation model on two transformed versions of a data point to reduce the difference between the outputs. SimCLR is a popular CL framework proposed recently, which trains a ResNet for latent features and an MLP for contrastive-loss computation [22]. Denote by x an input image patch and y the label of math or non-math. SimCLR aims to seek the optimal solution to

(1) $$arg\min_{G,F}L_{0}\left(G\left(F\left(T_{1}\left(x\right)\right)\right),G\left(F\left(T_{2}\left(x\right)\right)\right)\right)$$

where G is the MLP; F is the ResNet; x is a sample; $T_{1}$ and $T_{2}$ are two-time operators using the same family of augmentation; $L_{0}$ is the contrastive loss function, which is defined as

(2) $$L_{0}\left(z_{i},z_{j}\right)=-log\frac{exp(sim\left(z_{i},z_{j}\right)/\tau )}{\sum_{k=1}^{2N}1_{\left[k\neq i\right]}exp\left(sim\left(z_{i},z_{k}\right)/\tau \right)}$$

where *N* is the number of data points; τ denotes a temperature parameter; 1 is an indicator function; sim(u,v) where u and v are two input vectors [23]. However, the contrastive loss in Equation (3) fails to consider the subspace structure. That is, we in this study encouraged $z_{i}$ and $z_{j}$ to be in the same subspace such that the learned features are discriminative. We minimized the $l_{1}$-norm of the contrastive difference

(3) $$L_{1}\left(z_{i},z_{j}\right)={∥z_{i}-z_{j}∥}_{1}$$

where ${∥z∥}_{1}$ returns the maximal element in vector z. Once the maximal element was minimized in Equation (4), the $z_{i}$ and the $z_{j}$ have the most values on the exact coordinates and thus inhabit the same subspace. Therefore, this study used the following contrastive loss in our workflow,

(4) $$L=L_{0}+\rho L_{1}$$

where ρ is a trade-off parameter.

#### 2.2.2. Ensemble Classifier

After the stage of feature learning, we built an ensemble model to classify the images into math and non-math. MLP was employed to map an image feature to its label, where the image-slice label was given following the ground-truth label of the corresponding student. The used MLP aims to minimize

(5) $$\frac{1}{N}\sum_{i=1}^{N}∥H\left(z_{i}\right)-y_{i}{)∥}_{2}^{2}$$

where *N* is the number of samples; H denotes the MLP; z is the learned feature for x. However, each student has 20 image patches, leading to 20 labels. It is a multi-instance classification task. In this study, we considered all instances have the same importance to the student. We used the ensemble strategy to ensemble the 20 labels and voted for the label 0 or 1. The final predicted label is set to 1 if $sum_{k=1}^{20}l_{i}>10$ else 0, where $l_{i}$ is the predicted label for the *i*-th image instance.

### 2.3. Model Setting and Evaluation

The detailed setting in our workflow is as follows. In contrastive learning, the ResNet includes the layers by order: a convolutional layer with a kernel size of 3×3 from 1 to 64 channels, a residual module of 3 bottleneck blocks from 64 to 256 channels, a residual module of 4 bottleneck blocks from 256 to 512 channels, a residual module of 6 bottleneck blocks from 512 to 1024 channels, a residual module of 3 bottleneck blocks from 1024 to 2048 channels, and a final average pooling layer; the MLP for *G* includes two fully connected layers (2048-2048-128). The bottleneck block is composed of three convolutional layers with kernel sizes of 1, 3, and 1. Note that batch normalization is used following each convolutional layer and ReLU is used as the active function. In classifier training, the MLP has three layers where the numbers of neurons are 128, 64, and 1, respectively, and the activity function there is Sigmoid. These parameters of the used neural network model are the same as the original SimCLR [23] for comparisons. For our model, we set the parameter ρ=0.01 in Equation (5) for all experiments. Note that there is no extra balance parameter introduced into the used ensemble classifier.

In experiments, we partitioned the data into a training set and a test set by five-fold cross-validation. Specifically, we randomly partitioned the raw data into five subsets of roughly equal size. Four subsets were used as the training set, based on which we learned model parameters by 2000 iterations for contrastive learning and 1000 iterations for MLP learning. The remaining one subset was used as the test set, on which the learned classifier yielded 20 labels per student, and then the final result was reached by voting. The training and testing process was repeated five times such that each subset was used exactly once for validation. Accordingly, results are calculated on all 123 predictions.

This study evaluates the experiment results by calculating *ACC*, F1-score (*F*1), and *AUC*. From the confusion matrix, we first calculated the four metrics, i.e., True Positive (*TP*), False Positive (*FP*), False Negative (*FN*), and True Negative (*TN*). Then *ACC* and *F*1 are achieved by

(6) $$ACC=\frac{TP+FN}{TP+FP+TN+FN}$$

(7) $$Precision=\frac{TP}{TP+FP}$$

(8) $$Recall=\frac{TP}{TP+FN}$$

(9) $$F1=2\times \frac{Precision\times Recall}{Precision+Recall}$$

and *AUC* is defined as the area under the ROC curve that is plotted using *TP* and *FN* as the axis [27]. Besides, the two-tailed t-test for statistic significant test are also adopted to convince the classification result [28].

## 3. Results

### 3.1. Feature Visualization

**Figure 3:**
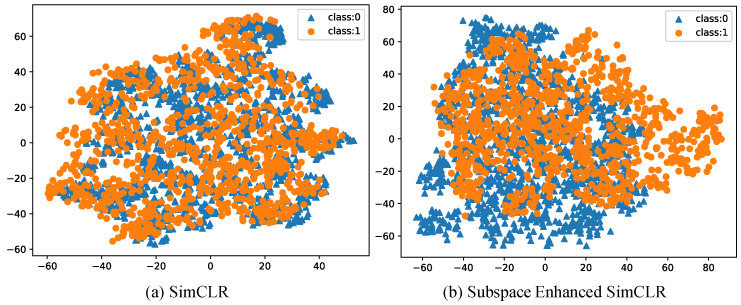
Visualization. 2D image features from SimCLR and the proposed Subspace Enhanced SimCLR are scattered in two subplots, respectively.

Figure 3 visualizes the image representations for 2D image patches, where the learned 2048 features are reduced into 50-dimensionality PCA subspace and then reduced into 2D t-SNE subspace. While implementing the t-SNE algorithm, we called the “TSNE” function in the “sklearn” package and set the perplexity value as 30.0, the default value. There are in total 2460 image representations, including 1440 images from math students (class 0) and 1020 images from non-math students (class 1). As is shown, the used Subspace-enhanced Simple framework for Contrastive Learning of visual Representations (SeSimCLR) yields more discriminative image representations than the original Simple framework for Contrastive Learning of visual Representations (SimCLR). We employed a one-layer perception to classify math students or non-math students on these 2D image representations, leading to an accuracy of 55.2% for SimCLR and 63.7% for the proposed method, respectively.

### 3.2. Overall Evaluation

Table 1 shows the evaluation results for image classification and student classification in accuracy (ACC), Precision, Recall, F1-score, and Area Under the Curve (AUC) of the Receiver Operating Characteristic (ROC) curve. We compared the two Contrastive learning (CL) models, i.e., SimCLR and SeSimCLR in Table 1, to validate the positive impact of the subspace enhancement on feature learning. On all metrics, SeSimCLR achieves significant improvements (*p*-values < 0.01) compared to SimCLR. These results show a low performance on the image classification, but the student classification arrived at high accuracy. This observation means that some image slices potentially suffer small impacts of math education and are thus hard to classify. It is possible to identify non-math students from math students using brain MRIs.

**Figure 4:**
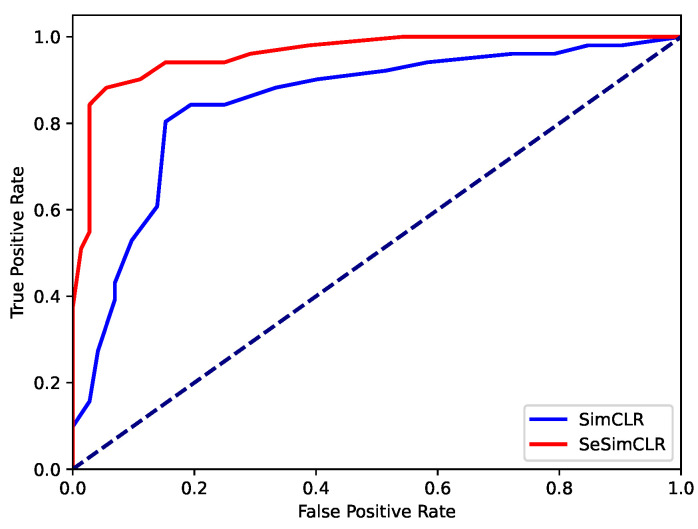
ROC curves. The ROC curves show the classification performance by the proposed workflow with SimCLR or SeSimCLR.

Figure 4 displays the ROC curves for student classifications on the 123 students by using SimCLR and SeSimCLR. For each student, we calculated the probability of the correct category by $N_{c}/20$, where $N_{c}$ is the number of correctly classified images for the target student. Then, using the ROCs in Figure 4, the AUCs in Table 1 were obtained. Both methods achieve decent AUCs, while SeSimCLR gains 0.14 improvement on AUC than SimCLR benefits from the subspace enhancement.

### 3.3. Detail Evaluation

**Figure 5:**
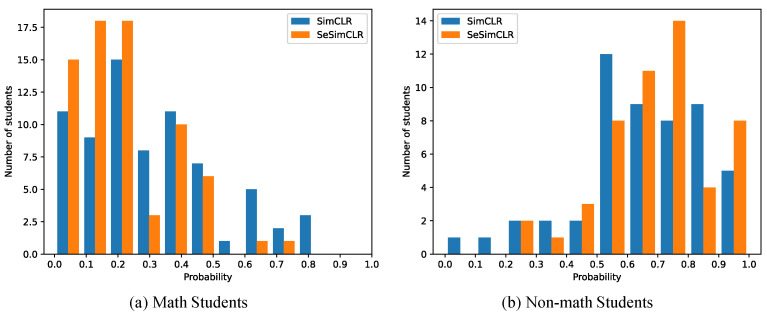
Histograms of classification probabilities. The number of students counts the students with the corresponding probability of belonging to class 1.

Figure 5 shows the number of students against the probability of students lacking math studying. This histogram distribution in Figure 5 shows the classification margin between the two categories, i.e., the math and non-math classes. Concretely, a student would be identified to the non-math class if the student had a greater than 0.5 probability. Otherwise, the student would be in the math class. On the one hand, from the statistical results in Figure 5, SimCLR identifies more math students with a greater than 0.3 probability than SeSimCLR, and meanwhile more non-math students with a less than 0.7 probability than SeSimCLR. On the other hand, for SimCLR, 35 of 72 students are classified as math class with a less than 0.3 probability. And 22 of 51 students are identified as a non-math class with a greater than 0.7 probability. While, for SeSimCLR, 51 of 72 students are identified as math students with a less than 0.3 probability. And 26 of 51 students are identified as non-math students with a greater than 0.7 probability. Therefore, the proposed SeSimCLR has a more considerable classification margin than SimCLR. Then the workflow with SeSimCLR yields a better classification performance in identifying non-math students from MRIs.

**Figure 6:**
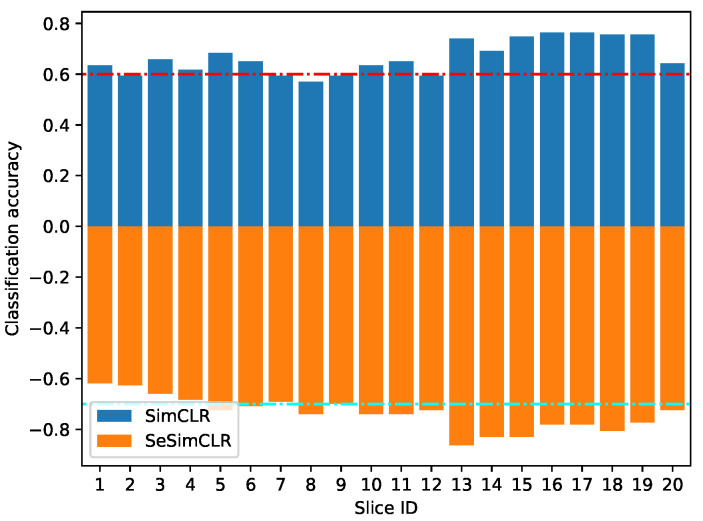
Classification accuracy on per image ID. The slice ID indicates the image id number of the 20 slices for each student. The negative probabilities were set to plot bars.

Figure 6 shows the image classification accuracy for each slice, where the slice ID varies from 1 to 20. This accuracy was calculated by the rate of corrected predicted images in all 123 images for each slice ID. As shown, there is high classification performance on the image slices from ID 13 to ID 19, while there is low accuracy on image slices ID 1, 2, 9, and 20. Besides, SeSimCLR achieves better classification performance than SimCLR on all slice IDs.

## 4. Discussion and Conclusions

In this paper, we attempted to identify whether a student lacks math education by using a machine learning model and student’s MRIs, where each student has 20 MRI image slices. Towards student classification, the proposed workflow consists of a CL model for feature learning, a MLP for MRI slice classification, and an ensemble voting for student classification. To improve the performance of SimCLR [23], we proposed to add a regularization item of subspace enhancement. That is to regularize the two representations of a sample into the same representation space.

The experiments were conducted on 123 students’ MRIs, including 51 math students and 72 non-math students. The commonly used metrics were employed to evaluate classification results at the level of image slices and the level of students, respectively. The results show that both SimCLR and SeSimCLR could yield favorable classification performance, resulting in an accuracy of about 70% for MRI slice classification and about 90% for student classification. Nevertheless, compared to SimCLR, SeSimCLR gains 7% and 5% improvements on MRI slice classification and student classification, respectively. Furthermore, the same conclusions could be reached regarding Precision, Recall, F1, and AUC. Hence, SeSimCLR could benefit from the strategy of subspace enhancement and achieve higher classification performance.

To further investigate the improvement from the use of SeSimCLR, we trained a classical CNN model [29] and the popular Residual Network (ResNet) model [30] on the raw 3D MRIs of size 20×20×20. To gain insight into the gains from the ensemble strategy, we obtained the features from SeSimCLR. We then concatenated the 20 feature vectors, training the CNN and the ResNet on the jointed features. With the same experiment settings, the student classification results by the four methods are listed in Table 2. From the results in Table 2, the classification performance benefits greatly from our workflow with SeSimCLR by comparing SeSimCLR with other methods. The ensemble strategy contributes significant improvements by comparing SeSimCLR with CNN(joint) and ResNet(joint).

**Figure 7:**
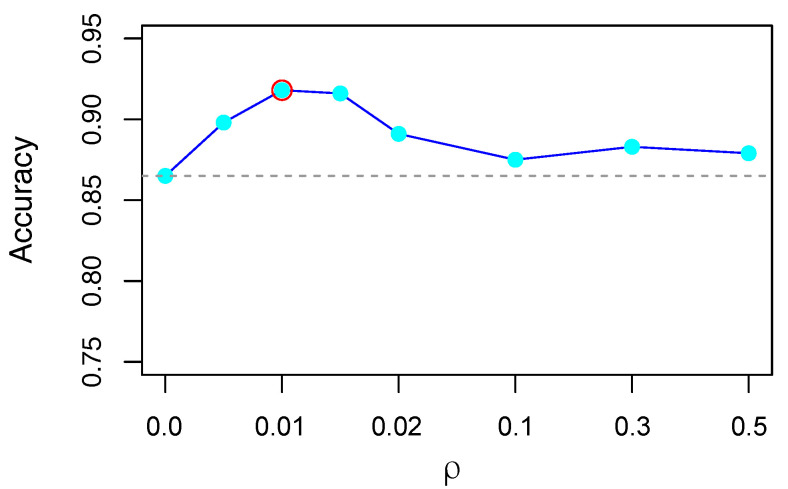
Accuracy against ρ. The classification results in terms of accuracy for various ρ.

To further investigate the sensitivity of the balance parameter, we conducted our experiments by varying ρ∈{0.0,0.01,0.02,01,0.3,0.5}. Figure 7 shows student classification accuracy against the parameter ρ in Equation (4). The results show that SeSimCLR achieves a relatively high accuracy (ACC) at nearby 0.01. The performance of SeSimCLR is consistently outperforming SimCLR when ρ lies in the range from 0 to 0.5. Hence, we set ρ=0.01 through all experiments.

From Figure 5 and Figure 6, two observations are worthy of mention. (1) The MRI slice classification has low performance due to the weak supervision from student labels. Still, the student classification is then successful by considering these classification results on 20 MRI slices per student. This observation means the ensemble classifier could lead to a better performance based on these weak base classifiers [24]. (2) The image slices have different classification accuracy, where several slices are easy in image classification. The observations potentially mean that the image slices of ID 13–19 were more impacted by math education. Besides, the subspace enhancement is effective for self-supervised deep feature learning in contrastive models.

Studies have shown that mathematics education is associated with IPS and MFG regions [3]. In our previous work [31], we proposed MiCL to study the influence of the intraparietal Sulcus (IPS) region on mathematics education. This work analyzes how the MFG area affects students’ mathematics education. The differences between the two methods are as follows. (1) In the MiCL method, we performed Non-math student prediction only on the level of students. The SeSimCLR method performs Non-math student prediction at both the student and image levels. (2) In the MiCL method, each student corresponds to a bag. There are 20 instances in a bag (i.e., 20 image slices corresponding to a student). Only the bag’s label is used to predict Non-math students without considering the label of each instance. In the SeSimCLR method, we use image slices to train 20 classification models. And an ensemble classifier is realized via voting for non-math student prediction. In general, the SeSimCLR method improves on the shortcomings of the MiCL method in non-math student prediction.

Some limitations of the proposed model and our study have not been reached. (*i*) In addition to the MFG region, other brain regions impact mathematics education. However, this study only uses the image data of the MFG region to identify non-math students. (*ii*) The pattern structure presented by the brain images can be explained by changes in the related molecules, so this study lacks such association analysis.

The future works would include: (1) integrating multi-region of the brain image data and employing the biomolecular data to reveal the internal mechanism of brain structure patterns that affect mathematics education. (2) learning the weight for each slice to improve the classification performance, since the image slices have various importance; (3) probing the open problem in deep-model parameter selection; (4) selecting the significant features by the promising DeepFeature model recently proposed by Alok et al. [32]; (5) integrating more brain region, like the intraparietal sulcus (IPS) [3]. In addition, the 3D deep classification model [21], and more data validation are also our future considerations.

## Figures and Tables

**Table 1:** Five evaluation indexes were calculated on all 123 students to compare the classification performance of SimCLR with SeSimCLR. Note that SeSimCLR is the used subspace-enhanced SimCLR.

	Images		Students	
	SimCLR	SeSimCLR	SimCLR	SeSimCLR
ACC	0.667	**0.737**	0.870	**0.918**
Precision	0.693	**0.788**	0.806	**0.972**
Recall	0.609	**0.626**	0.542	**0.619**
F1	0.648	**0.698**	0.648	**0.757**
AUC	–	–	0.947	**0.961**

**Table 2:** Classification results with the classical CNN model and the popular ResNet model trained on the 3D raw MRIs and the jointed features. All results were calculated on all 123 students.

Methods	Student Classification	
	ACC	AUC
SeSimCLR	**0.918**	**0.961**
CNN (3D)	0.772	0.857
ResNet (3D)	0.824	0.891
CNN (joint)	0.809	0.887
ResNet (joint)	0.849	0.923

## Data Availability

See https://github.com/ypzhaang/clr for the used codes and data (accessed on 24 June 2022).
